# College Students’ Knowledge of Ticks in Oklahoma: Assessment and Insights

**DOI:** 10.3390/insects12070658

**Published:** 2021-07-20

**Authors:** Elise Knowlton, Justin L. Talley, Bruce H. Noden, William Wyatt Hoback

**Affiliations:** 1 Department of Pediatrics, University of Oklahoma School of Community Medicine, Tulsa, OK 74135, USA; elise-knowlton@ouhsc.edu; 2 Department of Entomology and Plant Pathology, Oklahoma State University, Stillwater, OK 74078, USA; justin.talley@okstate.edu (J.L.T.); bruce.noden@okstate.edu (B.H.N.)

**Keywords:** tick biology, survey, college students

## Abstract

**Simple Summary:**

Knowledge of tick biology and how to prevent tick bites can help reduce the spread of tick-borne diseases; however, few studies have examined popularly held misunderstandings about ticks (e.g., incorrectly thinking that ticks can jump). To better understand tick knowledge, we surveyed college students before and after supplementary tick education. Tick knowledge after supplementary education improved, but important misunderstandings remained, such as ticks living in trees and Lyme disease being a concern in Oklahoma. This approach to determining misunderstandings through surveys and addressing misunderstandings via supplementary education may improve outreach and prevention programs aimed at raising tick-borne disease awareness as well as other programs seeking to offer arthropod education.

**Abstract:**

Ticks (Arachnida: Acari) are common in Oklahoma and may transmit tick-borne diseases (TBDs) to people. Due to the difficulty in reducing tick populations, awareness of tick bite prevention, proper tick removal, and knowledge of when to seek medical treatment are critical. However, outreach and extension programs are hampered by a lack of knowledge of what community members know about ticks. To address this limitation, we surveyed college students enrolled in three non-major Entomology courses at Oklahoma State University in 2018. Of the 483 students invited to take a survey, 224 (46.4%) students took both surveys. Pre-survey responses indicated lower levels of knowledge of tick biology compared to post-survey responses. For both pre- and post-survey respondents, “ticks can jump” and “ticks reside up in trees” received the *fewest* correct responses. A majority of survey respondents considered Lyme disease to be the predominant TBD in Oklahoma, although it is not established in Oklahoma. Supplemental education overcame these knowledge gaps, with the exception of knowledge of Lyme disease which was still considered to be the predominant TBD in the post-survey. Our results can be used to develop assessment tools to improve extension programs and enhance protection from TBDs.

## 1. Introduction

Ticks can transmit pathogens to humans, companion animals, and livestock. These pathogens include, but are not limited to, *Anaplasma* sp., *Babesia* sp., *Borrelia* spp., *Ehrlichia* spp., and *Rickettsia* spp. Tick-borne diseases (TBDs) account for the most human cases of vector-borne disease in the USA in the last two decades [1].

Ticks are documented from most Oklahoma counties with ranges of some species expanding geographically [2,3]. For example, the invasive eastern red cedar provides suitable habitat for the westward expansion in Oklahoma of *Amblyomma americanum*, the lone star tick [4]. Lone star ticks are associated with transmission of both *Ehrlichia chaffeensis* and *Ehrlichia ewingii* bacteria [5]. In 2017 in Oklahoma, 349 cases of spotted fever group rickettsiosis, 84 cases of ehrlichiosis, 41 cases of tularemia, and one case of Lyme disease were reported [6]. These numbers do not include unconfirmed cases. Furthermore, members of Oklahoma’s Native American tribes are diagnosed at a disproportionately higher rate with Rocky Mountain spotted fever and ehrlichiosis compared to other populations in the state [7,8,9]. For example, from 2001–2005, average annual incidence of Rocky Mountain spotted fever was 4.2 (per 1,000,000 persons) for white vs. 16.8 for American Indians [7]. Given that ticks are a healthcare burden with an estimated $712 million to $1.3 billion in total direct medical costs for the treatment of Lyme disease (and post-treatment of Lyme disease symptoms) alone, preventing tick bites is key to protecting human health [10].

Surveying community members to determine their existing tick knowledge and misunderstandings can guide and improve education and outreach programming. For example, in the Netherlands where Lyme disease is prevalent, a survey revealed social and practical barriers to tick bite prevention. When it is hot, citizens are less likely to wear long-sleeved protective clothing [11]. Additionally, citizens were found to be hesitant to apply insect repellent because of perceived risks of skin exposure to chemicals [11]. These barriers led researchers to develop education materials focused on proper removal of ticks rather than just preventing tick bites [11]. Misunderstandings about tick behavior may also exist, creating a false sense of security. For example, if one believes that ticks fall onto their hosts from a tree above, then solely avoiding woody habitats would produce a false sense of security; whereas, the real threat is near the ground where ticks are awaiting hosts to brush against them, a behavior known as questing. Ticks cannot jump but this misunderstanding also appears to be prevalent.

Ranchers of Oklahoma have been surveyed previously to assess their tick knowledge, attitudes, and practices (KAP). A majority of survey responses from beef producers across the state (*n* = 144, 78.7%) responded that Rocky Mountain spotted fever was their biggest concern [12]. Here we present a KAP survey administered to college students at Oklahoma State University at both the beginning and end of an academic semester. By surveying non-major college students, we explore what students know about aspects of tick biology and potential to transmit diseases. Second, we explore whether targeted supplementary education will overcome lack of tick knowledge or misunderstandings about ticks.

## 2. Materials and Methods

We used Qualtrics survey software to build and administer the survey (Survey Supplemental File) following the guidelines of the OSU Institutional Review Board. We administered the survey to three sections of two non-major Entomology courses: Insects and Society summer and fall sections taught by the same instructor, and Insects in Global Public Health taught by a different instructor.

For both the pre- and post-surveys, we generated a tick biology knowledge score for each student. We posed seven statements and questions (Table 1). For each correct answer, students received one point. We matched the same student’s beginning and end-of-semester surveys using anonymous codes that the students generated during the survey. We excluded responses from students who responded to only one survey.

We estimated levels of prior tick experience during the pre-survey by asking six questions at the beginning of the semester (Supplemental File). Students’ answers were scored as experience levels by assigning one point for each “yes” answer for up to 6 questions. For example, a student who responded “yes” to “Have you ever been bitten by a tick?” and “Do you know someone with a tick-borne disease?” had an experience level of 2.

We developed supplementary education to overcome students’ factual misunderstandings prior to post-surveys. The supplementary education was provided mid-semester to approximately 210 students attending the Insects and Society lecture in the fall course but not the summer course. The supplementary information contained an infographic about the life cycle of ticks [5] and an educational video (https://www.youtube.com/watch?v=_IoOJu2_FKE, accessed on 13 November 2018) by PBS Digital Studios’ Deep Look. After discussing the infographic and watching the video, students were prompted with the question, “Where did you see the ticks questing?” Next, students were instructed to answer two questions using the educational software, Top Hat (© 2019 Tophatmonocle Corp. Toronto, ON, Canada). The students were allowed to discuss answer choices prior to submitting responses.

We used SPSS version 26 (© IBM Corporation, Armonk, NY, USA) software for analysis. The Shapiro–Wilk test was used for assessing if variables (i.e., pre/post knowledge scores and experience level) approximated the normal distribution. Since data did not meet the normality assumption, the Median test (for independent groups) and Wilcoxon signed ranks test (for paired groups) were used in statistical comparisons of tick experience (across courses) and students’ pre/post knowledge scores respectively. Significance level was set at *p* < 0.05.

Given that supplementary education was directed to one of two sections of Insects and Society, we conducted a linear regression analysis to evaluate knowledge score change (post- minus pre-survey score) by course, controlling for demographic and other independent variables including race, ethnicity, gender, age, home state, course, and tick experience. For students preferring not to answer ethnicity and race demographic questions, and for one missing case (for race), these surveys were excluded from the model. Model assumptions were examined and satisfied prior to reporting the results of the model. Error term distributions were examined via histograms of standardized residuals and the normal probability plot. Multicollinearity was examined using variance inflation factors (VIFs), all below 1.36 (Mean: 1.21 ± 0.1). Homoscedasticity was examined via scatterplots of independent variables against the absolute value of residuals.

## 3. Results

### 3.1. Survey Response Rate

Students had the opportunity to complete the survey for extra credit. Table 2 summarizes response rates across academic year 2018 semesters and courses. Overall, 46.38% of students responded to both surveys (*n* = 224).

### 3.2. Demographics and Prior Tick Experience

**Figure 1:**
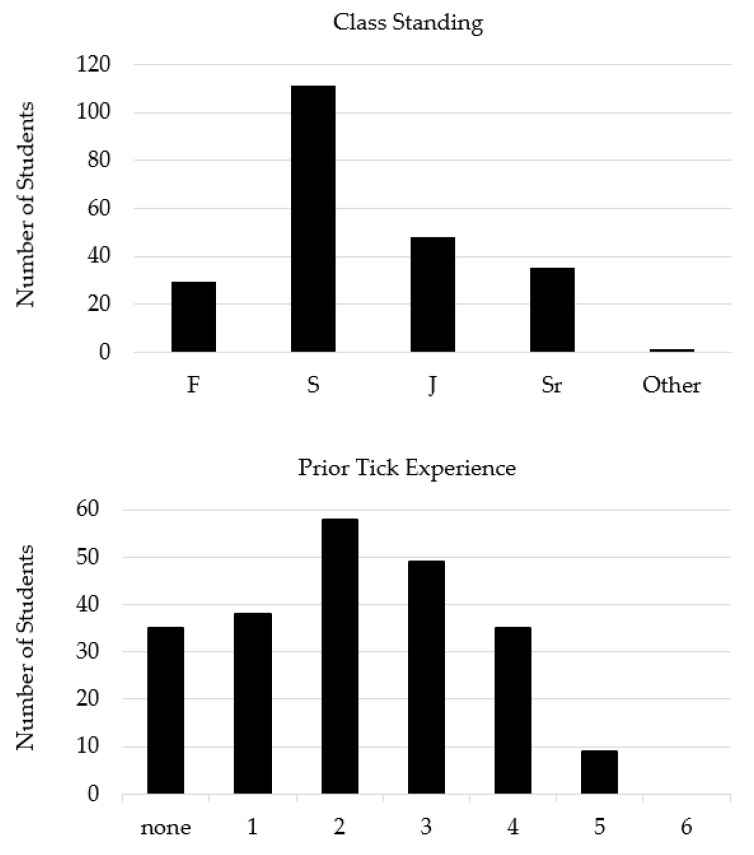
Class-standing (**Top panel**): F = Freshman, S = Sophomore, J = Junior, and Sr = Senior, and levels of prior tick experience (**Bottom panel**).

Half of pre- and post-survey respondents were sophomores (111/224 or 49.6%, Figure 1) with females in slight majority (122/224 or 54.5%). Survey respondents were between the ages of 18 and 37, with an average age of 19.8 years (±1.9 years, *n* = 224). Most survey respondents were White (*n* = 173) and non-Hispanic (*n* = 200). The top three majors of survey respondents were Management (15.6%), Finance (11.6%), and Media and Strategic Communications (8.9%). The top three home states of survey respondents were Oklahoma (62.5%), Texas (21.43%), and Illinois (2.68%). For survey respondents, the mean (±SD) tick experience was 2.17 ± 1.4 (Figure 1). Tick experience did not vary by course (Median test, χ^2^ = 0.347, *p* = 0.841); the median experience level was 2 for all courses: Summer Insects and Society (IQR:1–4), Fall Insects and Society (IQR:1–3), and Insects in Global Public Health (IQR:1–3). The tick experience of students was also examined in relation to observations of a tick on their pet. However pre-survey knowledge scores did not differ for students reporting ticks on pets from those who did not (Median test, χ^2^ = 1.645, *p* = 0.2).

### 3.3. Student Knowledge of Tick Biology

**Figure 2:**
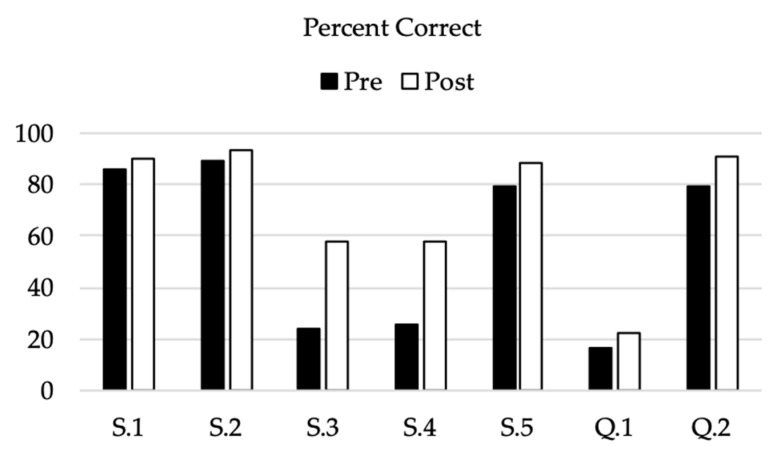
The percentages of correct responses to the seven statements and questions challenging knowledge of tick biology (*n* = 224). *X*-axis: abbreviated statements are “Ticks are in OK counties” (S.1), “Ticks may carry diseases” (S.2), “Ticks can jump” (S.3), “Ticks reside up in trees” (S.4), and “Ticks live in grass” (S.5). Question 1 asked which of five possible TBDs is most prevalent in OK, and question 2 challenged respondents to choose the image of a tick.

**Figure 3:**
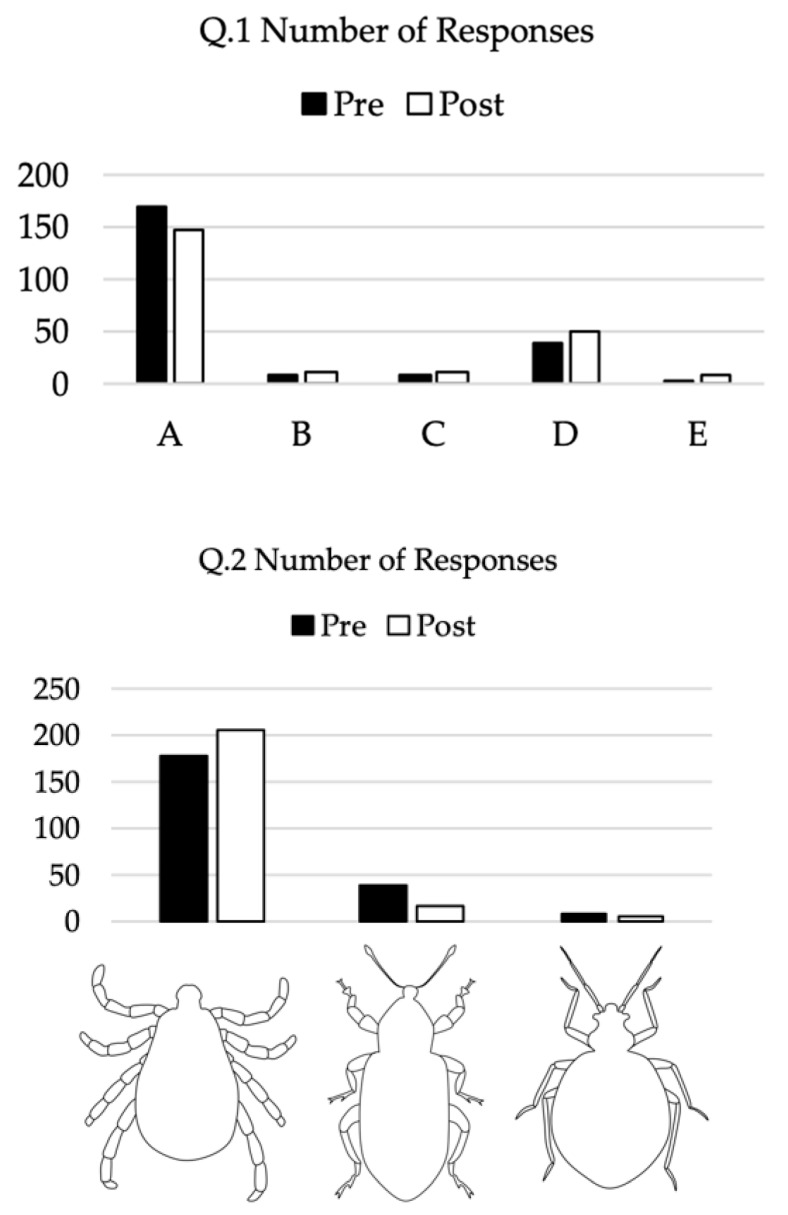
Top panel: Survey responses to “Which of the following is the most prevalent disease spread by ticks in Oklahoma?” (A = Lyme disease, B = Heartland virus, C = Ehrlichiosis, D = Rocky Mountain spotted fever, and E = Tularemia), and Bottom panel: Survey responses to “Which of the following is a tick?” (Image 1 = tick, Images 2 and 3 = insects).

On the pre-survey, we observed correct answers in a majority of responses to S.1 (85.7%), S.2 (89.3%), and S.5 and Q.2 (both 79.0% correct), and observed fewer correct responses to S.3 (24.1%), S.4 (25.9%), and Q.1 (16.5%, Table 3, Figure 2 and Figure 3). This pattern held for responses to the post-survey. S.3, S.4, and Q.1 had the fewest correct responses (Figure 2). Comparing each respondent’s pre- vs. post-survey knowledge score (defined as the total number of correct responses), we observed an increase of correct responses on the post-survey (Table 3, Figure 2). The median post-knowledge score was 5 (IQR:4–6) while the median pre-knowledge score was 4 (IQR:3–5), Wilcoxon signed ranks test, Z = −7.842, *p* < 0.001).

### 3.4. Supplementary Education

After delivering supplementary tick education to the fall semester course of Insects and Society (Table 2), the majority of students correctly answered in-class, fill-in-the-blank questions. For example, in response to “Ticks find their next host by…,” 92.8% of students (*n* = 192) correctly answered “crawling up onto blades of grass” and “attaching to a host that brushes up against them.” Likewise, 96.2% of students (*n* = 202) indicated “grass” and “vegetation on the ground” when prompted with “Ticks reside or live in…”.

By the end of the semester, we observed response differences between the two Insects and Society sections, summer (without supplementary education) and fall (with supplementary education). In a regression model of knowledge score difference (post- minus pre-survey scores, *n* = 215), students enrolled in the summer section had, on average, a reduced knowledge score difference of 0.78 compared to students enrolled in the fall section, holding all other variables constant (*p* = 0.014, Table 4 and Table 5).

## 4. Discussion

Studies assessing tick and TBD knowledge have focused on a variety of vulnerable populations, such as those at occupational risk for TBDs [12,13,14,15] and those facing linguistic and cultural barriers to TBD protection [16]. A survey of Brazilian immigrants living in a Lyme disease (LD) endemic area of the USA found that the majority of respondents (65%) had heard of LD; however, only a minority of respondents were very (4%) or somewhat certain (16%) they could identify LD symptoms [16]. Additional studies assessing general tick knowledge and awareness have shown discrepancies in responses by age [14,17,18] but see [19]. Respondents in older age groups in Missouri were more concerned with the threat of LD than younger age groups [17]. Similarly, respondents in Delaware who were 45 years and older were more likely to have heard of LD than respondents less than 45 years old [20]. In the present study, we surveyed college students typically among the age group scoring lower on tick knowledge compared to older age groups (e.g., 35–54 years, and 55+ years [18]). Little has been published on this younger, active cohort in the USA, especially in non-Lyme areas, where other tick species are more prevalent [5]. To the authors’ knowledge, this is the first survey that focused on what USA-based university students know about ticks.

Surveys ascertaining tick knowledge and awareness also typically differ in responses by region. The focal region of this study’s population was mainly from south central United States which answered differently from respondents in other published studies for other regions and countries. Studies of Canadian communities show significant spatial variation in LD knowledge [18,21,22], emphasizing the need for tailored educational approaches. In the USA, tick knowledge has been shown to vary along urban-to-rural gradients [17]. When Bayles et al. (2013) surveyed recreational park-goers in St. Louis, MO, the majority of respondents (99.2%) indicated some knowledge of ticks; however, only the minority could name a TBD other than Lyme. When examining survey responses more closely, the majority of rural park-goers could name a disease other than Lyme, while this was not the case for suburban and exurban park-goers [17]. However, even in a LD endemic region of the USA (Connecticut), the minority of survey respondents were aware that deer ticks carry pathogens causing diseases other than Lyme [19]. Ideally, educational programs should take tick population distributions and region-specific differences in tick awareness into account for developing effective education campaigns. Our study did not assess spatial variation in tick knowledge as most of the college students were from Oklahoma (*n* = 140) and Texas (*n* = 48). The observed levels of student knowledge were similar to those reported in different groups in other regions of the USA with higher awareness than those in the general population surveyed in St. Louis [17] and Delaware [20], but about the same for the national HealthStyles survey [23]. In the present study, the majority of students recognized that ticks are an issue in Oklahoma, that ticks transmit pathogens causing disease, and that ticks reside in particular habitats. Most also chose the correct image of a tick.

While the general awareness may be present, there is a critical lack of knowledge among university students in Oklahoma regarding tick biology, particularly in relation to habitats and their potential to transmit pathogens. Students in all three classes routinely answered that “Ticks can jump” (S.3) and “Ticks reside up in trees” (S.4) even after being taught otherwise by lecture or special supplemental material (Figure 2). Additionally, students were not aware about the most important tick-borne pathogens prevalent in the region, routinely answering ‘Lyme disease’ when there is no published record of *Borrelia burgdorferi* identified in *Ix. scapularis* ticks in Oklahoma [24]. Interestingly, this idea is common among the general public in the region with almost half reporting ‘Lyme’ as a major problem in the region [23]. This lack of knowledge and the apparent reticence in a majority to change or add to their knowledge even after being taught is a surprising result of this study.

There are several possible reasons for this lack of knowledge in Oklahoma-based university students. In a broad sense, a lack of exposure to outdoor activity or settings may possibly be a limiting factor in the experience of these students. Historically, most students attaining a Bachelor’s degree or higher are greater in urban than rural areas [25], which possibly means the urban students merely have not been exposed to outdoor risks. A St. Louis-based study reported that a majority of visitors to urban city parks were not concerned or aware of the risks of ticks and possible pathogens [17]. As such, they did not take the necessary precautions to prevent exposure. Additionally, there are also barriers limiting outdoor play in children and adolescents, particularly in urban areas [26], with screen technology quickly supplanting outdoor engagement [27]. When restrictive parenting limits outdoor play for young children [26], it is unknown if said restriction impacts outdoor activity patterns in adults. In addition, the percentage of children and adolescents (aged 11–15 years) rating connections to nature as important decreases as screen use increases [27]. When virtual media abound, including for learning, instilling (safe) connections to the outdoors may be a challenge.

Besides exposure, cultural beliefs also strongly influence understanding, particularly in changing preconceived ideas to scientifically-based facts [28,29]. A common cultural misunderstanding in Oklahoma is that if one were to find a tick on the head or scalp after being outdoors, it is because the tick fell there rather than crawling there. Studies have demonstrated that family beliefs strongly influence levels of acceptance for new scientific ideas [28,30]. The key is to try and engage students in ways that will reduce their shock at new ideas to help them reframe their cultural/family belief with methods to become more aware of the manageable risks around them.

While there is a great need for more detailed understanding, there is a basic level of knowledge regarding ticks that can be developed through more specialized teaching. In the case of this study, focused teaching in specific areas significantly contributed to changes in tick knowledge. When we provided supplemental education targeting gaps in tick knowledge, end-of-semester survey responses had significant, positive increases in correct responses to the most frequently missed questions, except for Q.1. involving tick-borne pathogens. Supplemental education included an informational video and an infographic with follow-up probing questions followed by an immediate in-class assessment. That day, post-lesson, most students correctly answered the tick questions. One of the limiting aspects of the study, however, is that we were not able to track for how long this change in response continues for these students.

One potential limitation to this study involved the population surveyed. Specifically, we administered the survey to a limited sample of college students at Oklahoma State University which may not be representative of all university students throughout the state and the geographic region. However, as the majority of students surveyed were either from Oklahoma or Texas, we feel their responses are representative of the region. We were also limited by the types of knowledge questions that could be asked in the context of a manageable survey. For example, we focused on tick habitat; however, tick biology and dispersal ecology are more expansive and complex in nature and difficult to capture in short question form. Lastly, the academic semester per se is also a limitation with only a few weeks to administer both surveys as well as the supplemental material. It also does not allow for the opportunity for long-term follow-up. However, it provides a solid baseline on which future studies can be developed.

## 5. Conclusions

In Oklahoma, ticks may be found along the entire urban-to-rural human land use gradient [31]. This, in combination with their high population sizes and pathogen transmission potential, make them important topics for education. The material delivered to college students during the academic semester improved overall tick knowledge, especially those aspects of tick biology that may be strongly held misconceptions. Given the potential for these misconceptions to influence protective practices, and the already well-documented regional discrepancies in tick knowledge around the USA, our findings further illustrate the need for specialized communication on ticks via targeted education. For the average college student in Oklahoma, becoming aware of pre-existing cultural beliefs and becoming informed on where ticks are, as well as what TBDs may affect them, can form the basis for effective educational initiatives. We suggest that future survey research focus on understanding ticks by exploring the misunderstandings of tick biology. These same approaches should apply to curriculum about insects and other arthropods where we believe that education can be improved by discovering and addressing misconceptions of the target audiences.

## Figures and Tables

**Table 1:** Statements/Questions challenging knowledge of tick biology.

No.	Statement/Question [Correct Answer]
S.1	Ticks are in a majority of Oklahoma counties. ^1^ [True]
S.2	Ticks in Oklahoma may carry diseases. ^1^ [True]
S.3	Ticks can jump. ^1^ [False]
S.4	Ticks reside up in trees. ^1^ [False]
S.5	Ticks live in the grass. ^1^ [True]
Q.1	Which of the following is the most prevalent disease spread by ticks in Oklahoma? ^2^
Q.2	Which of the following is a tick? ^3^

^1^ True/Not true/I don’t know answer choices. ^2^ Multiple choice answers, A–E: Lyme disease, Heartland virus, Ehrlichiosis, Rocky Mountain spotted fever (RMSF), or Tularemia [correct: RMSF]. ^3^ Multiple choice answers, images 1–3: Tick, Beetle, or Bed Bug [correct: Image 1, image is in Figure 3].

**Table 2:** Survey response rate by course.

Semester	Course	Pre- and Post-Respondents	Class (*n*)	Response (%)
Summer	Insects and Society	43	93	46.2
Fall	Insects and Society	126	266	47.4
Fall	Insects in Global Public Health	55	124	44.4

**Table 3:** Change in percentage of students with correct, incorrect, and I don’t know responses on the pre- and post-surveys (*n* = 224 paired respondents).

No.	Abbreviated Statement/Question	Correct Answer (%)	Incorrect Answer (%)	Don’t Know (%)
		(Post Minus Pre)	(Post Minus Pre)	(Post Minus Pre)
1	Ticks are in a majority of OK counties	4	3.1	−7.2
	(True, Not true, or I don’t know)			
2	Ticks may carry diseases	4	−1.3	−2.7
	(True, Not true, or I don’t know)			
3	Ticks can jump	33.9	−21	−12.9
	(True, Not true, or I don’t know)			
4	Ticks reside up in trees	31.7	−13.3	−18.2
	(True, Not true, or I don’t know)			
5	Ticks live in the grass	8.9	0	−9.3
	(True, Not true, or I don’t know)			
6	Most prevalent disease spread by OK ticks	5.8	−5.8	n.a.
	(Multiple choices A–E)			
7	Which of the following is a tick	12.1	−12.1	n.a.
	(Image: tick shape, insect shape, insect shape)			

**Table 4:** Descriptive statistics for variables used in OLS regression models of knowledge score difference for 224 college students.

Variable		Mean	SD	Range	Description
Dependent Variable					
Knowledge Score Difference		11.00	11.65	−4 to 7	Post- minus pre-survey score
Independent Variables					
Sex					
	Male	0.46	0.5	0 to 1	1 = male, 0 = female
Race					
	White	0.79	0.41	0 to 1	1 = white, 0 = else
Ethnicity					
	Hispanic	0.08	0.27	0 to 1	1 = Hispanic, 0 = else
Age		19.84	1.92	18 to 37	Patient age in years
Home State					
	Oklahoma (reference)	0.63	0.49	0 to 1	1 = Oklahoma, 0 = else
	Texas	0.21	0.41	0 to 1	1 = Texas, 0 = else
	Else	0.16	0.37	0 to 1	1 = else, 0 = OK and TX
Course					
	Fall Insects and Society (reference)	0.56	0.5	0 to 1	1 = fall section, 0 = else
	Summer Insects and Society	0.19	0.4	0 to 1	1 = summer section, 0 = else
	Insects in Global Public Health	0.25	0.43	0 to 1	1 = GPH, 0 = else
Tick Experience		2.17	1.41	0 to 5	Prior experiences’ score

**Table 5:** Coefficients from an OLS Regression Model of Knowledge Score Difference.

Variable	Unstandardized	Standardized	
	Coefficient	Coefficient	Significance
Sex	−0.053	−0.016	0.819
	(0.232)		
White	−0.074	−0.018	0.805
	(0.301)		
Hispanic	−0.495	−0.076	0.290
	(0.467)		
Age	0.060	0.065	0.383
	(0.069)		
Texas	0.239	0.060	0.444
	(0.312)		
Non-OK, Non-Texas	0.357	0.078	0.311
	(0.351)		
Summer Insects and Society	−0.782	−0.186	0.014 *
	(0.315)		
Insects in Global Public Health	0.138	0.036	0.621
	(0.279)		
Experience	−0.093	−0.079	0.322
	(0.094)		
Constant	0.121	0.930	
	(1.377)		

Notes: *n* = 215. Adj R^2^ = 0.015. Numbers in parentheses under the unstandardized coefficients are standard errors. * *p* < 0.05.

## Data Availability

For protection of human research subjects, only limited, fully de-identified data are available upon request.

## Supplementary Materials

The following are available online at https://www.mdpi.com/article/10.3390/insects12070658/s1, Supplemental File: Survey administered.
