# Supplementary material for: College Students’ Knowledge of Ticks in Oklahoma: Assessment and Insights
Source: Insects. 2021 Jul 20;12(7):658. doi: 10.3390/insects12070658 (PMC8303360; doi:10.3390/insects12070658)
Supplement: Supplementary file 1 [file insects-12-00658-s001.zip › insects-1226254-supplementary.pdf]

insects MDPI

Supplementary Material: College Students' Knowledge of Ticks in Oklahoma: Assessment and Insights

Survey Supplemental File:

Below we are going to provide some statements about ticks. Please select True, Not True, or I don't know.

1. Ticks are in a majority of Oklahoma counties.

- True
- Not True
- I don't know

2. Ticks in Oklahoma may carry diseases.

- True
- Not True
- I don't know

3. Ticks can jump.

- True
- Not True
- I don't know

4. Ticks reside up in trees.

- True
- Not True
- I don't know

5. Ticks live in the grass.

- True
- Not True
- I don't know

Next we are going to ask tick-related questions. Please select the answer or answers that seem most fitting.

6. Which of the following is the most prevalent disease spread by ticks in Oklahoma?

- A. Lyme disease
- B. Heartland virus
- C. Ehrlichiosis
- D. Rocky Mountain spotted fever
- E. Tularemia

7. Which of the following is a tick?

○ 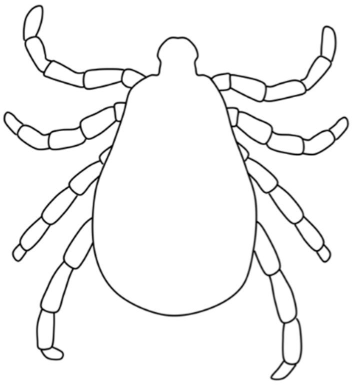

○ 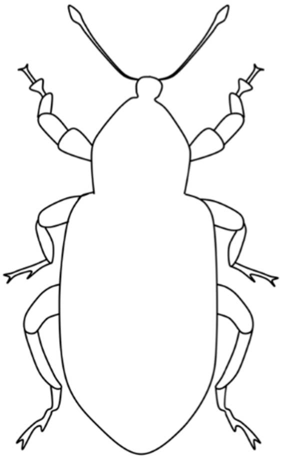

○ 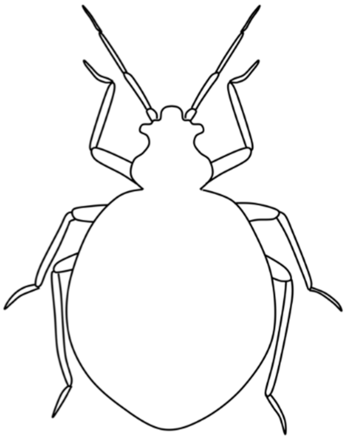

Below we are going to ask you some questions about any prior experience with ticks.

8. Have you ever seen a tick on your pet?

- ○ Yes
- ○ No
- ○ Not sure
- ○ Not applicable
- ○ Prefer not to answer

9. Do you check your pet for ticks after spending time outdoors?

- Yes
- No
- Not sure
- Not applicable
- Prefer not to answer

10. Have you ever been bitten by a tick?

- Yes
- No
- Not sure
- Not applicable
- Prefer not to answer

11. Have you ever seen a physician after being bitten by a tick?

- Yes
- No
- Not sure
- Not applicable
- Prefer not to answer

12. Do you know someone with a tick-borne disease?

- Yes
- No
- Not sure
- Not applicable
- Prefer not to answer

13. Have you ever developed a rash after being bitten by a tick?

- Yes
- No
- Not sure
- Not applicable
- Prefer not to answer
